# Postinfectious Acute Glomerulonephritis in Renal Transplantation: An Emergent Aetiology of Renal Allograft Loss

**DOI:** 10.1155/2019/7438254

**Published:** 2019-03-18

**Authors:** Alícia Molina-Andújar, Enrique Montagud-Marrahí, David Cucchiari, Pedro Ventura-Aguiar, Erika De Sousa-Amorim, Ignacio Revuelta, Frederic Cofan, Manel Solé, Adriana García-Herrera, Fritz Diekmann, Esteban Poch, Luis F. Quintana

**Affiliations:** ^1^Department of Nephrology and Renal Transplantation, Hospital Clínic, University of Barcelona, IDIBAPS, Barcelona, Spain; ^2^Department of Pathology, Hospital Clínic, University of Barcelona, Barcelona, Spain

## Abstract

Despite the high incidence of posttransplant infections, postinfectious acute glomerulonephritis (PIAGN) in renal allograft is a rare entity, without effective treatment and a bad prognosis. We describe two cases of PIAGN: the first one was developed 2 years after kidney transplantation, secondary to* Staphylococcus aureus* bacteremia with presence of extracapillary proliferation in biopsy. The patient was treated with methylprednisolone and plasma exchanges without response, remaining dialysis dependent. The second case was reported 5 years after kidney transplantation, secondary to influenza A infection. Kidney biopsy showed an IgA-dominant PIAGN and methylprednisolone boluses were initiated without clinical response, suffering a progressive worsening and loss of kidney graft. Due to the aggressive clinical course of this entity, PIAGN should be considered in the differential diagnosis of acute kidney graft failure in the context of an infection. Elderly patients have a higher risk of more severe acute renal dysfunction, requiring dialysis in a great proportion of cases.

## 1. Introduction

PIAGN is a reactive immunological process which involves the kidney following a nonrenal infection. Poststreptococcal glomerulonephritis is a classic example, but there are other infections that can trigger this glomerular injury [1–4]. In adults, PIAGN is more common in immunocompromised patients, particularly older, diabetics, and alcoholics [5]. In renal allograft, it is a rare entity leading to graft loss in many cases [1].

Recently, a new form of PIAGN characterized by IgA-dominant immunocomplex deposits has been described [6]. Its histological and ultrastructural features resemble classical PIAGN but in which IgA is the dominant or codominant Ig found in glomerular immune deposits.

We describe two cases of renal graft loss with an unusual origin, related to PIAGN of offbeat course that depict a new perspective in the clinical approach of renal recipient with nephritic syndrome and reflect the current changes in the patient profile with ESRD characterized by a continual aging with an associated higher susceptibility to infections under immunosuppressive therapy.

## 2. Case Presentation

### 2.1. Case 1

A 69-year-old man affected with IgA nephropathy received his third deceased-donor kidney transplantation in 2015 after two previous grafts were lost due to primary nonfunction. Immunosuppression consisted of thymoglobulin induction, tacrolimus, mycophenolate, and prednisone. Baseline serum creatinine was 1,5 mg/dl with normal urinalysis.

**Figure 1:**
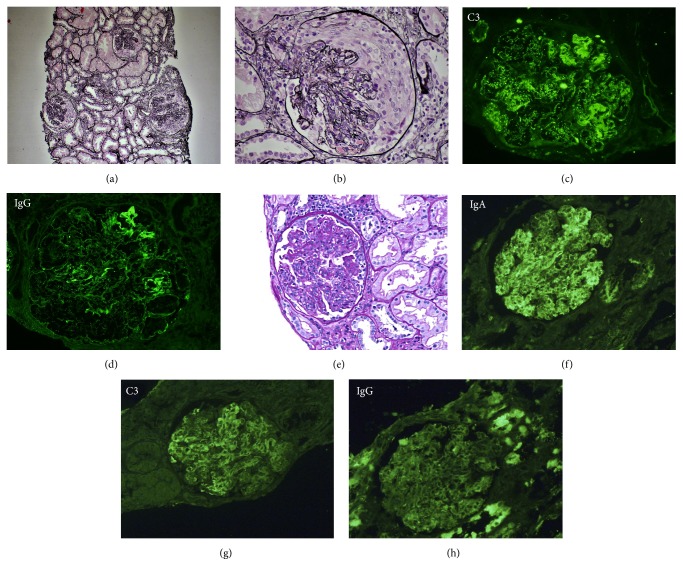
*Case 1* (a-d): (a), (b) diffuse alteration of the glomerular structure with marked endocapillary inflammatory hypercellularity, double contours, and crescent formation. Silver stain. (c) Immunofluorescence C3 (+++) with granular pattern and mesangial and capillary distribution. (d) Immunofluorescence IgG (+++) with granular pattern and mesangial and capillary distribution.* Case 2* (e-h): (e) glomerulus with marked endocapillary inflammatory hypercellularity. PAS stain. (f) Immunofluorescence IgA (+++) with granular pattern. (g) Immunofluorescence C3 (++) with granular pattern and mesangial and capillary distribution. (h) Immunofluorescence IgG (-).

In November 2017, he was admitted to hospital because of fever with positive blood culture for* Staphylococcus aureus*. He was treated with cloxacillin plus daptomycin and endocarditis was ruled out with a transesophageal echocardiography, with negative control blood cultures. After six days of treatment he developed a nephritic syndrome with hemodialysis requirement. Immunofixation and autoimmunity tests were negative, but a C3 consumption was observed. A kidney biopsy showed diffuse alteration of the glomerulus structure with marked endocapillary inflammatory hypercellularity, double contours, and crescent formation. Immunofluorescence was positive for C3 and IgG. It was compatible with PIAGN with endocapillary and extracapillary proliferation (Figure 1). Donor Specific HLA-Antibodies were not detected. The patient was treated with three pulses of methylprednisolone (500 mg/day) and plasma exchanges without response, remaining dialysis dependent. A new biopsy showed chronicity signs, with fibrous capsular thickening, fibrous crescents, and retracted glomeruli.

### 2.2. Case 2

A 65-year-old man with end-stage renal disease (ESRD) secondary to diabetic nephropathy received his first deceased-donor kidney transplantation in 2013. Immunosuppression consisted of thymoglobulin induction, tacrolimus, mycophenolate, and prednisone. Baseline serum creatinine was 1 mg/dl with normal urinalysis.

On January 31^st^, 2018, he was hospitalised due to Herpes Zoster Virus meningoencephalitis, treated with acyclovir for 2 weeks with good response. Having no symptomes of encephalitis, he suffered from influenza A infection on February 10^th^, treated with oseltamivir. Six days later that infection, he developed a nephritic syndrome with dialysis requirement. Immunofixation and autoimmunity tests were negative, but a C3 consumption was present. Donor Specific HLA-Antibodies were not detected. A graft biopsy showed glomerular endocapillary inflammatory hypercellularity and reactive hyperplasia. Immunofluorescence was positive for IgA and C3 (Figure 1) and IgA-dominant PIAGN diagnosis was made. Three methylprednisolone pulses (250 mg/day) were administrated without response and need for chronic hemodialysis.

## 3. Discussion

*De novo* glomerulonephritis after kidney transplantation is a potential cause of graft dysfunction, being PIAGN a plausible example of this emerging clinical scenario. However, PIAGN in transplant recipient is a rare entity with only 16 cases previously reported [1–4]. Infection focuses and causative agents are also changing: not only* streptococcal* but also other microorganisms can trigger a PIAGN [1].

PIAGN usually presents with a nephritic syndrome with C3 hypocomplementemia. Classical histological findings are diffuse endocapillary proliferation with frequent subepithelial “humps” and subendothelial deposits. Immunofluorescence typically shows dominant granular C3 and mesangial IgG deposits [1, 6].

Surprisingly, the first recognition of infection usually coincides with the onset of signs and symptoms of glomerulonephritis, suggesting that many infections are initially subclinical. It is extremely important not to delay graft biopsy in this clinical context to improve treatment response.

In 2003, a morphological variant of classical PIAGN called IgA-dominant PIAGN was described [7]. Its main histological finding is the presence of dominant or codominant granular and mesangial deposit of IgA. It is a very rare variant that typically occurs in diabetic 60-year-old males after a skin and soft tissue infections by* S. aureus *[2, 6–8].

In kidney transplant recipients, there is only one reported case of IgA-dominant PIAGN. In this regard our case is, to our knowledge, the second one reported of IgA-dominant PIAGN in a kidney recipient and the first with a virus as a trigger [2, 6].

It is fundamental to differentiate this novel entity from the classical IgA nephropathy triggered by an S aureus infection using the following criteria [9]. In addition to an IgA mesangial deposit (major criteria), there should be at least one of the following: (a) endocapillary proliferation, (b) hypocomplementemia, or (c) subepithelial deposits (electron microscopy) [6]. Present case met these diagnostic criteria.

There is no specific treatment in the PIAGN: current strategies rely on culture-guided systemic antibiotics, support therapy, and steroids associated with plasma exchange when crescents are present [10–12]. Despite that treatment, graft loss occurs in up to 60% of cases [1]. In IgA-dominant PIAGN involving native kidneys, reported prognosis despite treatment is equally poor: 16% present a complete recovery, 49% have partial renal dysfunction, and up to 38% progress to ESRD [6]. In the only case published to date on a kidney graft, the authors report a favorable outcome [2]. On the contrary, our patient with IgA-dominant PIAGN progressed to ESRD. Perhaps, because we had to use lower methylprednisolone doses (250 mg/d x 3) than in the case reported by Anand et al. [2], since our patient had presented a recent meningoencephalitis.

Over the last decades, the renal transplant waiting list has grown significantly older: in the United States, between 1997 and 2014, the proportion of ≥65-years-old candidates grew from 7 to over 21% [13]. Although the PIAGN is an uncommon cause of graft loss, transplant clinicians should be aware of aging and the immunodeficiency degree because immunosuppression and recipient comorbidities could increase the frequency of PIAGN [14].

In conclusion, we present 2 cases of renal graft loss secondary to PIAGN, highlighting the first reported case of IgA-dominant PIAGN secondary to a viral infection in a kidney transplant recipient. Due to its aggressive clinical course, it should be considered in the differential diagnosis of acute kidney graft failure with nephritic syndrome in the context of an infection. Elderly patients have a higher risk of infections and therefore the aging of the waiting list could increase its incidence [13]. Moreover, prognosis in renal allograft is worse than in native kidney. More studies are needed to provide an in-depth knowledge of PIAGN and to define an effective therapeutic approach.

## Data Availability

The clinical, laboratory, and pathology data used to support the findings of this study are included within the article.
